# Effect of Solid-State Fermentation of *Hericium erinaceus* on the Structure and Physicochemical Properties of Soluble Dietary Fiber from Corn Husk

**DOI:** 10.3390/foods13182895

**Published:** 2024-09-12

**Authors:** He Ban, Qiannan Liu, Lin Xiu, Dan Cai, Jingsheng Liu

**Affiliations:** 1 College of Food Science and Engineering, Jilin Agricultural University, Changchun 130118, China; bh121318@163.com (H.B.); lqn0511@163.com (Q.L.); jluxiulin1979@sina.com (L.X.); liujingsheng@jlau.edu.cn (J.L.); 2 National Engineering Research Center for Wheat and Corn Deep Processing, Changchun 130118, China

**Keywords:** *Hericium erinaceus*, corn husk, solid-state fermentation, fermentation kinetics, dietary fiber modification, structure and function

## Abstract

Corn husk, a by-product of corn starch production and processing, contains high-quality dietary fiber (DF). Our study compares and analyzes the impact of *Hericium erinaceus* solid-state fermentation (SSF) on the structure and physicochemical characteristics of soluble dietary fiber (SDF) of corn husks. The study also investigates the kinetics of SSF of *H. erinaceus* in this process. The scanning electron microscopy (SEM) and Fourier transform infrared spectroscopy (FT-IR) results revealed significant structural changes in corn husk SDF before and after fermentation, with a significant elevation in the functional group numbers. The data indicate that the fermented corn husk SDF’s water-holding, swelling, and oil-holding capacities increased to 1.57, 1.95, and 1.80 times those of the pre-fermentation SDF, respectively. Additionally, the results suggest that changes in extracellular enzyme activity and nutrient composition during SSF of *H. erinaceus* are closely associated with the mycelium growth stage, with a mutual promotion or inhibition relationship between the two. Our study offers a foundation for corn husk SDF fermentation and is relevant to the bioconversion of maize processing by-products.

## 1. Introduction

Corn husk constitutes a rich dietary fiber (DF) by-product of corn processing and production. The husk contains approximately 60 to 70 percent of corn DF, which can absorb up to 1.5 g of water [1]. This high water absorption capacity can elevate feces volume, accelerate their transportation in the digestive tract, and improve constipation [2]. Meanwhile, corn husk DF has been found to have various physiological functions, including lowering blood glucose and lipids [3], reducing weight [4], improving intestinal flora [5], and preventing cancer [6]. These functions have gained attention recently due to their potential impact on human health. DF can be divided into insoluble dietary fiber (IDF) and soluble dietary fiber (SDF) according to their solubility. SDF primarily comprises gums, pectins, oligosaccharides, and soluble hemicellulose. In contrast, IDF mainly includes lignin, cellulose, and insoluble hemicellulose [7]. Corn husk is a rich source of IDF and SDF. However, the proportion of SDF in corn husk DF is low (0.2–2.6%) [8], which results in poor water solubility, palatability, and, consequently, limited absorption and utilization by the body. Furthermore, incorporating corn husks may exert a deleterious influence on food products, particularly in the context of dough [9]. Excessive levels of IDF within the gluten matrix are inimical to the formation of the gluten network, which ultimately diminishes the dough’s elasticity and causes a concomitant reduction in product quality [9]. As a result, corn husk DF tends to be utilized in feed manufacturing or discarded, leading to resource waste and environmental pollution [10]. Previous studies have demonstrated that SDF exhibits superior biological activity and physiological functions than IDF [11]. Therefore, modifying corn husk DF can improve its extraction rate, enhance its physiological activity, increase the comprehensive utilization rate of corn, save resources, and promote social and economic benefits. Furthermore, corn husk is widely available at a low cost [12], and modified corn husk dietary fiber can be produced on an industrial scale.

Previous research has demonstrated that microorganisms can modify DF through fermentation, which converts certain IDF in the raw material to SDF. This process also enhances DF’s structure and physicochemical properties, making it more advantageous for human health and more straightforward. Fungi solid-state fermentation (SSF) represents a microbial modification technique enhancing DF utilization. This method offers several advantages, including a short production cycle, low cost, mass production, and environmental friendliness [13]. The fungi commonly used for cellulase production are mainly molds, such as *Rhizopus oryzae* [14], *Trichoderma viride* [15], *Monascus anka* [16], and *Aspergillus niger* [17]. Some fungi produce enzymes such as protease, amylase, and cellulase during growth, such as *Pleurotus ostreatus* [18] and *Ganoderma lucidum* [19]. Studies have shown that *Kluyveromyces marxianus* fermentation significantly increased the DF content, hydration properties, and adsorption capacity. The microstructure of the DF surface showed a loose and porous structure, indicating a reduction in the degree of polymerization of cellulose [20]. According to Si [17], DF treated with *Aspergillus niger* fermentation exhibited high oil retention capacity and nitrite ion, cholesterol, and sodium cholate adsorption.

*Hericium erinaceus* (*HE*) is an edible medicinal fungus that contains various nutrients, including proteins, cellulose, polysaccharides, peptides, mannitol, terpenoids, and flavonoids [21]. It also has physiological functions, such as antioxidation [22], anti-aging [23], treatment of gastric ulcers [24], and neuroprotection [25]. *HE* can secrete many extracellular enzymes during growth and fermentation due to its complete enzyme system [26]. During *HE* fermentation, these enzymes can hydrolyze lignin, cellulose, hemicellulose, proteins, and starch components of corn husk [27]. The resulting small nutrient molecules are absorbed and utilized by the mycelium, increasing the content and purity of corn husk SDF. A previous study utilized *HE* SSF to modify corn husk DF and concluded that this process could enhance corn husk SDF content and functional characteristics [28]. However, knowledge regarding the *HE* mechanism of action in corn husk fermentation demonstrated in the above study is limited. Therefore, this study employed the SSF of *HE* to analyze the fermentation influence on corn husk SDF structure and physicochemical properties comparatively. Additionally, the study investigated the correlation between extracellular enzymes and variations in associated nutrients throughout SSF. A kinetic analysis of SSF by *HE* was also conducted to enhance the fermentation of modified DFs and corn husk SDFs.

## 2. Materials and Methods

### 2.1. Samples and Chemicals

Corn husks procured from Huanglong Food Industry Co., Ltd. (Jilin, China) were rinsed utilizing deionized water until reaching a neutral pH value, dried in a constant temperature oven at 50 °C (101A-1ET, Shanghai Experimental Instrument Factory Co., Ltd., Shanghai, China), ground with a pulverizer (AISITE, Tianjin Taiste Instrument Co., Ltd., Tianjin, China), sieved through a 0.25 mm mesh screen, and followed by −20 °C storage until the next step of determination.

The strain *HE-02-06* was purchased from the National Engineering Centre for Deep Processing of Wheat and Maize. Moreover, we procured sodium carboxymethylcellulose, xylan, and L-tyrosine standards from Shanghai Yuanye Biotechnology Co., Ltd. (Shanghai, China) and glucose standards from Tanmo QC-Standard Material Centre (Beijing, China). Casein was provided by Beijing Auboxing Biotechnology Co. (Beijing, China). The other chemicals were of analytical purity.

### 2.2. Fermentation Strain Cultures

The strain was cultured using the solid slant method based on the previous technique [29], with slight modifications. The conserved *HE* strain was inoculated onto 12°. Malt agar medium was incubated at a constant temperature for 7 d in a 27 °C incubator (HPX-9162MBE, Shanghai Boxun Industrial Co., Ltd. Medical Equipment Factory, Shanghai, China) before being set aside.

The strain liquid activation medium was based on the earlier outlined composition and content [30] with certain alterations, which contained 30 g of anhydrous glucose, 10 g of yeast dipping powder, 20 g of soluble starch, 3 g of potassium dihydrogen phosphate (PDP), and 0.6 g of magnesium sulfate heptahydrate (MSH) per liter. The pH was natural, and the medium was sterilized for 20 min at 121 °C. The solid slant strain was cultured and inoculated with 30 mL liquid activation medium in 100 mL conical flasks. The first-grade strains were obtained by constant temperature oscillation culture for 6 d at 27 °C and 160 r/min using an oscillation incubator (Stab S2, Shanghai Rundle Biotechnology Co., Ltd., Shanghai, China). Secondly, the primary fermentation strain went through inoculation into 500 mL conical flasks that contained 200 mL of liquid activation medium and were cultured under persistent temperature oscillation at 27 °C and 160 r/min for 6 d to obtain the secondary fermentation strain.

### 2.3. SSF of Corn Husks

The SSF method was adopted from the previous method [31], with minor modifications. The SSF medium was prepared at a 1:2 ratio using 15 g of corn husk powder and 30 mL of distilled water, followed by adding 1.4 g of anhydrous dextrose, 0.05 g of PDP, and 0.02 g of MSH. The medium was sterilized for 20 min at 121 °C. The medium was inoculated with the secondary fermentation strain of *HE* and incubated for 7 d at a fermentation temperature of 25 °C and an inoculum volume of 80 mg/mL. Once the fermentation process was complete, the solid fermentation medium was lyophilized at −80 °C, ground with a pulverizer, sieved through a 0.25 mm mesh screen, and stored at −20 °C.

### 2.4. Preparation of Corn Husk SDF 

A mixture of corn husk powder before and after fermentation and distilled water was prepared at a 1:20 g/mL ratio, immersed in a water bath (DK-98-II, Tianjin Tester Instrument Co., Ltd., Tianjin, China), and maintained at 80 °C for 2 h to extract the desired components. During this procedure, agitation was achieved by employing a vortex mixer (QL-901, Jiangsu Haimen Qilin Medical Instrument Factory, Haimen, China). Afterward, the solution was cooled and centrifuged using a low-speed centrifuge (LD5-2B, Beijing Leibel Centrifuge Co., Ltd., Beijing, China) at 4000 rpm for 20 min. After centrifugation, the supernatant was treated with four volumes of 95% ethanol, causing the development of precipitates. These precipitates were freeze-dried for 48 h, following a 12 h storage at 4 °C. Our study assessed the fermentation broth’s DF content, employing the enzyme weight method specified in the Chinese national standard GB/T 5009.88-2014.

### 2.5. Structural Characteristics of Corn Bran SDF

#### 2.5.1. Scanning Electron Microscopy (SEM) 

Observation of the corn husk SDF microstructure was conducted using SEM (Ge miniSEM, ZEISS, Jena, Germany) according to Du [32], alongside slight adjustments to the procedure to obtain SEM images. The sample stage was coated with conductive adhesive, onto which an appropriate quantity of SDF samples was evenly dispersed and secured. A gold layer was uniformly sprayed over the samples, and their morphology was examined under SEM at 12–15 kV at various magnifications (5000× and 20,000×).

#### 2.5.2. Fourier Transform Infrared Spectroscopy (FT-IR) 

With minimal modifications, SDF molecular structure and chemical groups were analyzed prior to and following fermentation using a method analogous to that Gan [33] described. The background was provided by using blank potassium bromide. The transmittance of SDF was evaluated using a Fourier transform infrared spectrometer (VERTEX70, Shanghai Experimental Instrument Factory Co., Ltd., Shanghai, China) throughout a range of scanning wavelengths from 400 to 4000 cm^−1^.

### 2.6. Corn Bran SDF Physicochemical Characteristics 

#### 2.6.1. Water-Holding Capacity (WHC)

The determination of the WHC was carried out according to Zhang, Y. [34] with slight adjustments. More precisely, a dried SDF sample weighing 0.2 g was measured and introduced into a 15 mL centrifuge tube with 10 mL of distilled water. After a comprehensive blending process, the mixture was set for 24 h at room temperature (RT) and centrifuged for 10 min at 5000 rpm. The supernatant was then poured out, measuring the remaining residue weight and calculating WHC as follows:

$$\mathrm{WHC}(g/g)=(m_{1}-m_{0})/m$$

where *m* (g) represents the sample mass, *m*_0_ (g) represents the centrifuge tube and sample mass, and *m*_1_ (g) represents the centrifuge tube and sample mass after aspiration.

#### 2.6.2. Water Soluble Capacity (WSC)

The WSC was measured following Zhang, M. [35], with little variation. In short, we placed 0.2 g of a dried SDF sample in a 10 mL measuring cylinder. The dried sample volume in the natural stacked state was recorded, followed by adding 7 mL of distilled water, stirring gently, and leaving at RT for 24 h to permit the sample to settle. The SDF sample volume after absorbing and dissolving water was recorded, calculating WSC as follows:

$$\mathrm{WSC}(\mathrm{mL}/g)=(V_{1}-V_{0})/m_{0}$$

where *m*_0_ (g), *V*_0_ (mL), and *V*_1_ (mL) reflect the sample mass, the sample volume, and the sample volume following water absorption and swelling, respectively.

#### 2.6.3. Oil-Holding Capacity (OHC)

The OHC was evaluated following Caprez’s method [36], with slight adjustments. Briefly, a dried SDF sample weighing 0.2 g was measured and moved into a 15 mL centrifuge tube. Afterward, 10 mL of soybean oil was introduced into the tube, mixed thoroughly, and left to sit at RT for 2 h. The sample was then centrifuged at 5000 rpm for 10 min. The uppermost layer of oil was meticulously extracted, and the mass of the remaining residue was ascertained, calculating the OHC as follows:

$$\mathrm{OHC}(g/g)=(M_{1}-M_{0})/M$$

where *M* (g), *M*_0_ (g), and *M*_1_ (g) refer to the sample mass, the centrifuge tube and sample mass, and the centrifuge tube and the sample mass after oil absorption, respectively.

### 2.7. Determining Nutrient Content

#### 2.7.1. Total Starch

The total starch content in the fermentation broth was quantified through the enzymatic method outlined in the Chinese national standard GB/T 5009.9-2016 [37].

#### 2.7.2. Reducing Sugar

The Johnson et al. [38] methodology for quantifying the concentration of reducing sugars was adjusted with alterations specifically for this investigation. At first, a precise amount of 1 g of corn husk powder was measured and combined with 10 mL of distilled water. The solution was thereafter extracted in a water bath for 10 min and maintained at 80 °C. After the extraction process, the substance was cleaned with distilled water in a flask with a volume of 25 mL. It was then filtered, and 5 mL of the filtered substance was moved into another flask with a volume of 25 mL and filled to the top. Afterward, 1 mL of the sample solution was transferred by a pipette into a 10 mL centrifuge tube, and distilled water was added to reach a 2 mL final volume. Then, 4 mL of DNS reagent was introduced into the tube, heating the sample solution in a water bath at boiling temperature. Once the solution was cooled to the temperature of the surrounding room, the optical density (OD) value was assessed at a 540 nm wavelength after calibration and shaking. The quantification of decreasing sugar content in the fermentation broth was subsequently assessed through these measures. 

#### 2.7.3. DF

Our study assessed the DF content in the fermentation broth employing the enzyme weight method specified in the Chinese national standard GB/T 5009.88-2014 [39].

### 2.8. Determining Extracellular Enzyme Activity 

#### 2.8.1. Extraction and Preservation of Crude Enzyme Solution (CES)

The CES was acquired using a revised procedure derived from the methodology described by Daou, C. [40]. At first, 20 mL of a 0.05 mol/L citrate buffer solution with a pH of 4.6 was introduced to 2 g of the solid medium that had undergone fermentation. The combination was subsequently extracted in a water bath consistently maintained for 2 h at 30 °C. Following the extraction process, the mixture was subjected to centrifugation using a high-speed centrifuge (Allegra X-30R, Beckman Inc., Indianapolis, IN, USA) at 8000 rpm for 15 min at 4 °C. The liquid portion formed after spinning at high speed was meticulously gathered and distributed into EP tubes and then kept at −80 °C for future utilization.

#### 2.8.2. Amylase 

Amylase activity was assessed via an adjusted method described originally by Hussien, S. A. [41]. The experiment commenced with the combination of a 0.5 mL solution of the crude enzyme, which had been diluted fivefold and warmed for 15 min at 40 °C, with 1 mL of a preheated soluble starch solution (10 mg/mL, 0.1 mol/L citrate buffer soluble, pH 5.6). The reaction occurred in a water bath maintained at 40 °C for 30 min, followed by adding 2 mL of 3,5-dinitrosalicylic acid (DNS) reagent, heating the water bath for 5 min, and subsequently cooling. The glucose concentration was quantified by determining the OD at 520 nm after diluting the solution to 25 mL. The CES that was rendered inactive was used as the control group. Enzyme activity was quantified in units per milliliter (U/mL), determining amylase activity as follows:

$$U=\frac{C\times V\times W\times 1000}{t\times V_{1}}$$

where *C* (mg/mL) is the glucose concentration in the reaction system, *V* (mL) represents the reaction system total volume, *W* represents the CES dilution factor, 1000 represents the conversion factor of glucose mg to μg, *t* (min) represents the reaction time, and *V*_1_ (mL) represents the amount of enzyme involved in the reaction.

#### 2.8.3. Carboxymethylcellulase (CMC) Enzyme

The method used for detecting CMC enzyme activity was based on Sarangthem Indira’s approach [42] with modifications. A mixture of 0.5 mL of a fivefold diluted CES and 1.5 mL of a 5 mg/mL sodium CMC enzyme solution (prepared with 0.1 mol/L, pH 4.6 acetate buffer) was incubated for 30 min in a 50 °C water bath, followed by adding 2 mL of DNS. The mixture was boiled in a water bath for 5 min before being cooled to stop the reaction. The resulting mixture was fixed to 25 mL, and the OD value was determined at 540 nm to calculate the glucose concentration. The inactivated CES was used as the control group. Enzyme activity was expressed as U/mL, and CMC enzyme activity was calculated as follows:

$$U=\frac{C\times V\times W\times 1000}{t\times V_{1}}$$

where *C* (mg/mL) is glucose concentration in the reaction system, *V* (mL) represents the reaction system total volume, *W* represents the CES dilution factor, 1000 represents the conversion factor of glucose mg to μg, *t* (min) represents the reaction time, and *V*_1_ (mL) represents the amount of enzyme involved in the reaction.

#### 2.8.4. Hemicellulose (HC) Enzyme 

According to the procedure outlined by Christoph Ottenheim [43], with adjustments made for the detection of HC enzyme activity, 0.5 mL of a CES diluted five times and preheated at 50 °C for 5 min was mixed with 1 mL of a xylan solution (10 mg/mL). The xylan solution was made by combining 0.1 mol/L pH 4.6 acetate buffer, which had been preheated to 50 °C for 5 min, with the combination being subsequently incubated for 30 min in a 50 °C water bath. Afterward, 2 mL of DNS reagent was introduced, heating the combination for 5 min in a water bath prior to being allowed to cool to stop the reaction. Subsequently, the mixture was diluted to 25 mL, determining the xylose concentration by measuring the OD value at 540 nm. The CES that was rendered inactive was used as the control group. Enzyme activity was quantified in units per milliliter (U/mL), determining HC enzyme activity as follows:

$$U=\frac{C\times V\times W\times 1000}{t\times V_{1}}$$

where *C* (mg/mL) is xylose concentration in the reaction system, *V* (mL) represents the reaction system total volume, *W* represents the CES dilution factor, 1000 represents the conversion factor of xylose mg to μg, *t* (min) represents the reaction time, and *V*_1_ (mL) represents the amount of enzyme involved in the reaction.

### 2.9. Statistical Analysis

The experiments were replicated three times, reporting the findings as the average value plus or minus the standard deviation. The differences between means were evaluated using one-way analysis of variance (ANOVA). *p* < 0.05 indicated a statistically significant level. The data were analyzed using IBM SPSS Statistics 23 (IBM Corporation, Armonk, NY, USA) and GraphPad Prism 8.0 (San Diego, CA, USA), which were used to create plots as well as graphical representations.

## 3. Results and Discussion

### 3.1. SEM Analysis

**Figure 1:**
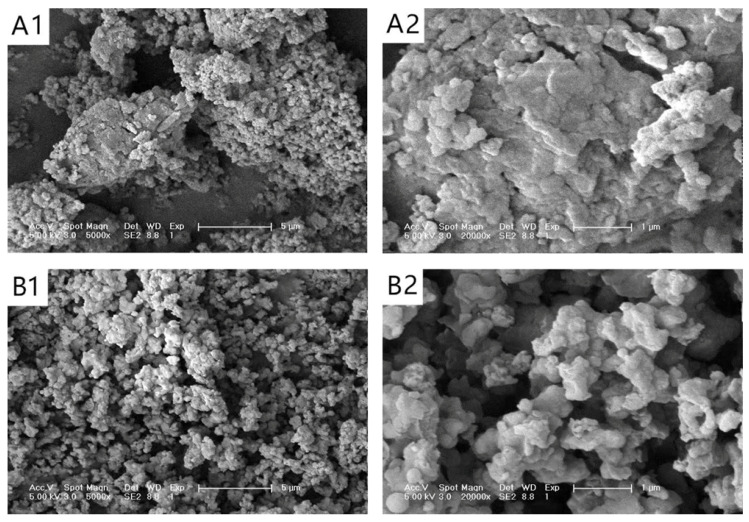
Scanning electron microscope (SEM) images depict soluble dietary fiber (SDF) before fermentation and after fermentation. (**A1**) SDF pre-fermentation at ×5000 magnification; (**A2**) SDF pre-fermentation at ×20,000 magnification; (**B1**) SDF post-fermentation at ×5000 magnification; (**B2**) SDF post-fermentation at ×20,000 magnification.

Figure 1 illustrates the SEM images of corn husk SDF before and after fermentation. The pre-fermentation corn husk SDF particles were larger and had an uneven size; irregular, flaky surface; and dense structure (Figure 1(A1,A2)). In contrast, the post-fermentation corn husk SDF particles, as shown in Figure 1(B1,B2), were smaller and more uniform in size, with irregular bumps on the surface and a honeycomb structure. The surface area of the post-fermentation corn husk SDF had significantly increased, and there were significantly more small, loose particles than the pre-fermentation corn husk SDF. These results align with prior studies [44]. The above phenomenon may be attributed to the hydrolysis of metabolites produced during the fermentation of the strain, such as enzymes like CMC and HC, which break the macromolecular glycosidic bonds of the cellulose long chain [45], converting IDF to SDF. At the same time, the original SDF structure in the maize hulls is altered, decreasing molecular weight, polymerization degree, and SDF particle size. In addition, the change in the SDF-specific surface area causes a more robust water–oil binding capacity [46].

### 3.2. FT-IR Analysis

**Figure 2:**
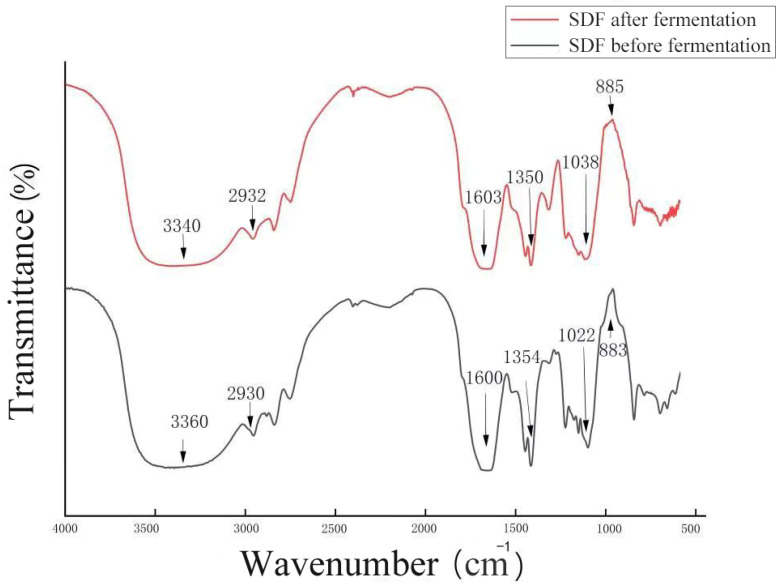
Fourier transform infrared (FT-IR) spectroscopy of SDF before and after fermentation.

Figure 2 depicts the FT-IR spectra of corn husk SDF pre- and post-fermentation in the 400–4000 cm^−1^ region. The broad peak near 3300 cm^−1^ resulted from the O-H bond stretching vibration from the hydroxyl group of cellulose and HC bound to hydrogen [47]. The presence of this broad peak indicates the presence of free hydroxyl groups in SDF. The absorption peak at approximately 2930 cm^−1^ results from the C-H bond stretching vibration in polysaccharide methylene groups, which is typical of cellulose absorption peaks [11]. The absorption peak near 1600 cm^−1^ may be formed because of the characteristic absorption of C=O bonds by glyoxalate [15], and the width of this peak in the SDF of fermented maize hulls widens in a small amount. The absorption peak at 1030 cm^−1^ is due to the stretching vibration of the C-O bonds in cellulose and HC [16]. This peak is typical of the absorption peak of arabinoxylan [48]. The intensity of this peak is greater after fermentation, indicating that the fermented maize husk SDF contains more arabinoxylan. The absorption peak near 1350 cm^−1^ was attributed to the C-H bond bending vibration [49]. The absorption peak near 880 cm^−1^ was that of the β-glycosidic bond, and the intensity of this peak was reduced after fermentation, which was likely due to the breaking of the glycosidic bond during the fermentation process [49]. The absorption peaks of corn husk SDF did not significantly change before and after fermentation in terms of their characteristic types. The functional groups remained basically the same, indicating that the types of hydrophilic groups and some reactive groups in SDF were not altered. However, there was a slight change in the width, intensity, and wave number of the absorption peaks, which may be because of the hydrolysis throughout fermentation that destroyed the molecular structure of the DF and exposed the functional groups.

### 3.3. Physicochemical Properties of Corn Husk SDF Pre- and Post-Fermentation

**Figure 3:**
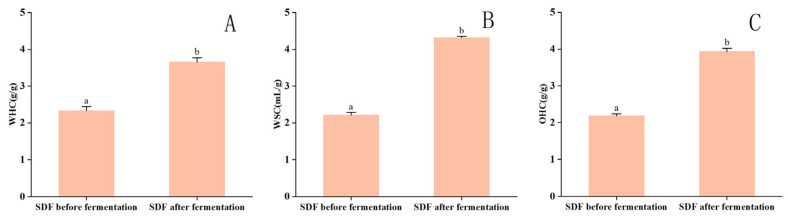
Comparison of physicochemical properties of corn husk SDF pre- and post-fermentation. Diverse lowercase letters reflect statistically significant differences between treatment groups (*p* < 0.05). (**A**) Water-holding capacity (WHC) of SDF before and after fermentation; (**B**) expansion capacity (WSC) of SDF before and after fermentation; (**C**) oil-holding capacity (OHC) of SDF before and after fermentation.

Figure 3 depicts the WHC, WSC, and OHC of corn husk SDF pre- and post-fermentation.

WHC is the water quantity that can be held by 1 g of dry fiber during particular circumstances of soaking time, temperature, and centrifugation time and speed [50]. Figure 3 shows that the WHC of corn husk SDF increased by 1.57 times to 3.65 ± 0.12 g/g after fermentation compared to the pre-fermentation period. Fermentation modification increased the SDF’s ability to retain water, which may be due to an elevated SDF content and the honeycomb structure [51]. In addition, the WHC of DF exhibited a close correlation with SDF particle size, surface properties, and source [52].

WSC refers to the ability of fibers to absorb water when immersed in it. The WSC of fermented corn husk SDF increased by 1.95 times compared to pre-fermentation, reaching 4.31 ± 0.04 mL/g, as shown in Figure 3. FI-IR (Figure 2) revealed that the breaking of β-glycosidic bonds of cellulose and HC exposed more hydrogen bonds, thereby increasing the WSC [53]. This phenomenon may also be related to the increased SDF content [54]. Research has demonstrated a negative correlation between the WSC of DF and its particle size. Specifically, reduced particle size increases the specific DF surface area, increasing WSC [51].

OHC refers to the amount of oil retained after centrifugation of fiber mixed with oil. It is a significant attribute of DF. This ability might potentially affect the absorption of dietary lipids in the intestines, hence playing a role in regulating body weight and aberrant blood lipid levels. The OHC of corn husk SDF increased 1.80-fold after fermentation compared to the pre-fermentation period, reaching 3.93 ± 0.09 g/g (Figure 3). The increased OHC may be because of the production of a porous and loose structure of SDF during fermentation. Additionally, the increased SDF content was accompanied by increased content of pectin, arabinoxylan, and other components. These components can enhance the adsorption and removal of both saturated and unsaturated lipid materials because of their intense attraction to lipid materials, increasing OHC [55].

To summarize, the fermentation-modified corn husk SDF exhibited a significant elevation (*p* < 0.05) in WHC, WSC, and OHC, possibly due to the hydrolytic effect of microbial metabolism throughout fermentation. This effect results in smaller corn husk SDF particles, increasing the contact area of SDF with water and oil. After fermentation, the structure of SDF is disrupted, exposing more groups and increasing SDF binding sites. As a result, the WHC, WSC, and OHC of corn husk SDF are increased. This result is consistent with the findings of Fan et al. [56], who demonstrated that fermented bran DF has a more remarkable ability to bind water and assist in the formation of a gluten network, which improves the strength of gluten in noodles as well as the performance of the noodles. It can, therefore, be concluded that fermentation-modified DF can further improve the quality of food products. Concurrently, the augmented water-holding capacity of modified DF in food enhances human intestinal health and facilitates optimal digestion and defecation [57]. Furthermore, its augmented oil-holding capacity can assist in the reduction of fat absorption, the regulation of body weight, and the enhancement of cardiovascular health [20]. In conclusion, the incorporation of modified DF in food products can enhance satiety and reduce overall calorie intake [58]. Nevertheless, the introduction of modified dietary fibers may result in alterations to the taste and texture of food products [59], which may not meet consumer expectations. It is, therefore, necessary to make technological advances in order to improve these undesirable changes. Furthermore, many consumers lack familiarity with modified dietary fibers and may be skeptical of the concept of ‘modified’. Consequently, the capacity to accurately convey the advantages of modified DF on product labels is a crucial factor influencing consumers’ purchasing decisions. In conclusion, further research and refinement could be conducted in the future for food products enriched with fermentation-modified corn husk DF.

### 3.4. Analysis of Changes in Nutrient Composition

The total starch content had an overall reduction trend throughout *HE* growth, proving that the carbon nutrients were gradually absorbed and utilized. The most significant decrease in starch content was observed in 0–6 d of fermentation, and the decrease in the middle and late fermentation stages was relatively slight and leveled off, fluctuating around 3.27%. This phenomenon may be due to the rapid growth of *HE* in the early fermentation stage; the mycelium produced significant lipid and protein amounts by using starch during the growth process, decreasing the total starch content [60]. The growth of *HE* in the late stage of fermentation tends to stabilize or begin to age, and the nutrients in the medium are more abundant, so the use of starch is reduced.

*HE* growth and development are inextricably linked to reducing sugar content. As a nutrient that can be directly absorbed by edible fungal mycelium, changes in the content of reducing sugars have an essential effect on the development of edible fungal substrates [61,62]. During fermentation, the reducing sugar content showed an overall decreasing trend, which increased significantly during 0–2 d of fermentation and peaked at 7.04 mg/g on the 2nd day. During 2–6 d, the reduced sugar content decreased significantly and extensively on the 6th day, followed by increasing and then decreasing. Then, it decreased to the lowest value of 0.56 mg/g on the 10th day and leveled off. The change in reducing sugar content during fermentation can reflect the change in CMC and HC enzyme’s vitality [27] as well as the conversion between IDF and SDF [63]. In the early fermentation stage, IDF was hydrolyzed to generate a large amount of reducing sugar to meet the nutritional needs of mycelial growth; the amount of reducing sugar generated was greater than the amount consumed to be accumulated. The reduced sugar content gradually decreased in the later stages of fermentation, and these results were attributed to the following three factors: the depletion of reducing sugars by the fermentative growth of *HE* mycelium, the limited enzyme vigor, and the inhibitory effect of the enzyme by the enzyme degradation products [64]. 

**Figure 4:**
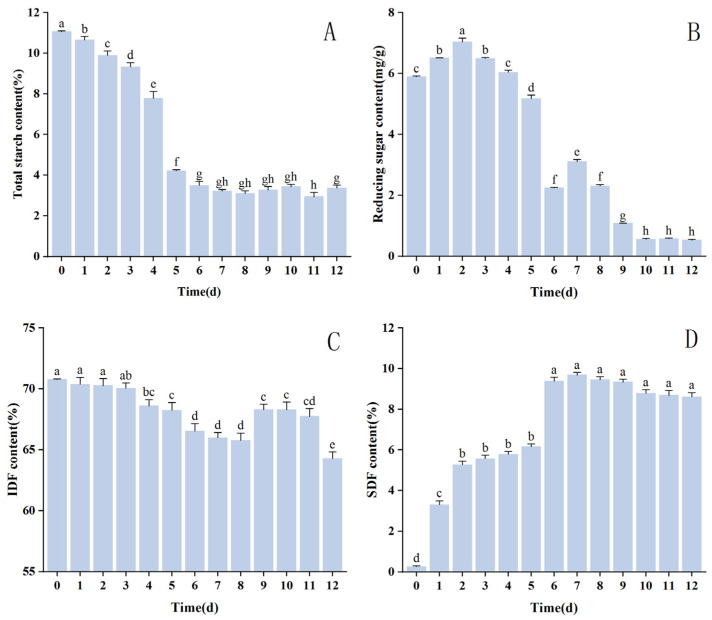
Alterations in nutrient composition of corn husk SDF pre- and post-fermentation. Dissimilar lowercase letters signify statistically significant alterations between treatment groups (*p* < 0.05). (**A**) Changes in total starch content; (**B**) changes in reducing sugar content; (**C**) changes in insoluble dietary fiber (IDF) content; (**D**) changes in SDF contents.

Figure 4 shows the changes in IDF and SDF contents during fermentation. The IDF content exhibited a gentle decline overall, remaining almost unchanged in the early stages of fermentation before gradually decreasing with time. On the 12th day of fermentation, the IDF content in the medium decreased to 64.29% of the raw material. The content of SDF exhibited an early rise followed by a slow decline. From the 6th to the 7th of fermentation, the SDF content in the medium remained stable at approximately 9.38 g/100 g before gradually decreasing. The above changes indicate that IDF was converted to SDF during fermentation. Enzymes such as amylase, produced by the strain during the pre-fermentation period, hydrolyzed the chemical bonds connecting SDF with components such as starch and proteins, thereby improving the purity and content of SDF [65]. As the minor molecule nutrients in the medium were consumed, the enzymes such as CMC and HC produced during fermentation decomposed the IDF and SDF in corn husk to produce minor molecule nutrients such as oligosaccharides, disaccharides, and monosaccharides for the growth of the bacterium, gradually decreasing IDF and SDF contents. However, due to the gradual aging of the mycelium in the late fermentation stage, the ability to absorb nutrients was weakened, so the decrease in IDF and SDF was not significant.

Since Figure 4 shows that nutrient content changes during fermentation, different optimal fermentation times can be determined depending on the desired application. For example, for products targeting low-carbohydrate diets, such as low-carbohydrate pasta substitutes, the fermentation time can be adjusted to maximize certain nutritional benefits while reducing the carbohydrate content. As Figure 4 illustrates, the total starch and reducing sugar content of corn husk modified with *HE* fermentation was decreased significantly on the sixth day, which is a beneficial characteristic for those following a low-carbohydrate diet. Furthermore, total starch and reducing sugar content changed very little during the subsequent fermentation period, suggesting that an excessively long fermentation period is not overly beneficial to the fermentation environment and wastes resources. In conclusion, the sixth day of fermentation represents the optimal timeframe for the production of low-carbohydrate diet products. 

Conversely, for the production of products like noodles, the texture and nutritional content of the noodles need to be considered. According to Figure 3 and Figure 4, the fermentation with *HE* improves the nutritional value and physicochemical properties of the corn husk, such as a reduction in the crude fiber content, an increase in the SDF content, and the enhancement of the properties, such as the WHC of the corn husk’s SDF. All of these changes contribute to the production of noodles with better taste and nutritional properties [56]. By combining Figure 4 with the socio-economic factors, it can be determined that the seventh day of fermentation, when the IDF content is lower and the SDF content is higher, represents the optimal fermentation time for making noodles.

To conclude, further research could be conducted to enhance the performance of corn husk fiber in specific applications by optimizing the fermentation process. This may be for the production of pasta with a specific texture and nutritional composition or the development of products suitable for low-carbohydrate diets. The combination of control of the fermentation process and adjustment of nutritional parameters can result in a notable enhancement of the quality of specific food products.

### 3.5. Changes in Extracellular Enzyme Activity

**Figure 5:**
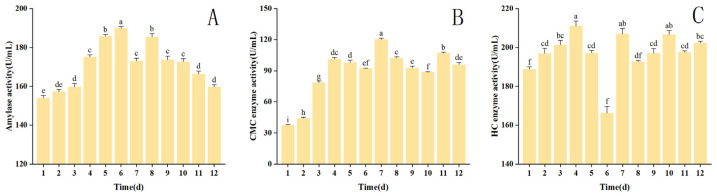
Variations in extracellular enzyme activity of corn husk SDF pre- and post-fermentation. Dissimilar lowercase letters denote statistically significant differences between treatment groups (*p* < 0.05). (**A**) Changes in the viability of amylase; (**B**) changes in the viability of Carboxymethylcellulase (CMC) enzyme; (**C**) changes in the viability of Hemicellulose (HC) enzyme.

The changes in the viability of the amylase, CMC, and HC enzymes during fermentation are shown in Figure 5.

Fungi can use the extracellular enzymes secreted by their fermentation to degrade organic macromolecules into smaller units and obtain nutrients for their growth [66]. Therefore, changes in extracellular enzyme vigor correlate with changes in nutrient content within the substrate and also respond to the development of fungal seed bodies.

The amylase activity during fermentation exhibited an early surge followed by a subsequent decline, indicating an overall trend. Over the first 6 days of fermentation, the activity of amylase significantly rose (*p* < 0.05), reaching its peak value of 189.90 U/mL on the sixth day. Amylase activity decreased from day 8 to day 12, with the lowest activity recorded at 159.69 U/mL on the 12th day. *HE* utilized amylase to metabolize starch from nutrients in the solid medium as a carbon source, decreasing starch content during this process. The high amylase enzyme activity during the pre-fermentation period may be attributed to the rapid growth of the strain. The decrease in amylase enzyme activity during the later stage may be due to the cessation or overgrowth of the strain, as well as the unavailability of nutrients [67,68].

The activity of the CMC enzyme increased during the pre-fermentation period and significantly rose from day 1 to day 4 of fermentation (*p* < 0.05). The activity peaked at 120.18 U/mL on the seventh day, after a decrease in activity from day 4 to day 6, and then declined from day 7 to day 10. The CMC enzyme activity in Monascus moniliensis remained low throughout the fermentation process. The data show that the CMC enzyme has low activity during the fermentation process of *HE*, consistent with previous research [69]. The lower cellulose enzyme activity indicates weaker cellulose degradation by *HE*. This phenomenon may occur because cellulose degradation is ranked lower than HC degradation. If hemicellulose degradation provides sufficient nutrients for the growth of *HE*, then it does not require a large amount of cellulose degradation to provide nutrients for its growth. Although the CMC enzyme exhibited low overall enzyme activity, it demonstrated high activity during its late fermentation stage, consistent with a previous study [70].

Similarly, during the pre-fermentation period, the activity of the HC enzyme exhibited a significant growth trend, reaching a peak of 211.04 U/mL on day 4. Subsequently, it declined on days 4–6, reaching a minimum on day 6, before increasing significantly on day 7. It then declined on day 8, followed by slow growth on days 9–10. The trend of HC enzyme activity has been observed in many previous studies. Keisuke Tokimoto [71] found that the activity of the HC enzyme reached its maximum during the maturation of *shiitake mushroom* substrates. Similarly, Takao Terashita [72] discovered a comparable regularity. HC enzyme activity gradually increased during the growth of *reishi* mycelium, with peak enzyme activity occurring at the maturity of the substrate [73]. The studies above demonstrate that the HC enzyme exhibits a specific growth pattern in edible mushrooms. The enzyme activity peaks at the substrate’s maturity, indicating a correlation between the HC enzyme and the development of the edible mushroom substrate.

The activity of the three extracellular enzymes in the fermentation process showed an overall trend of increasing and decreasing. A significant change occurred on the 7th and 10th days of fermentation. The strains mainly grew and reproduced at the early fermentation stage, producing low extracellular enzyme activity. As the growth of the strains stabilized, the enzyme activity improved. Amylase activity is related to utilizing substrates by microorganisms [74]. Figure 5 shows that the changes in amylase activity were similar to the trend of nutrient composition in Figure 4, which supports the notion that amylase activity is related to substrate concentration. Overall, the HC enzyme activity was higher than that of the CMC enzyme. This phenomenon supports the idea that HC is degraded before cellulose in the degradation order. Simultaneously, the activity trends of the CMC and HC enzymes were found to be more similar. This phenomenon reflects the correlation between the changes in cellulase and HC enzyme activities. Changes in one enzyme’s activity may affect the activity of the other enzyme [75]. The study demonstrated that the enzymes CMC and HC exhibited activity peaks during the early stage of fermentation for protoplast formation and the late stage of fermentation for harvesting. This occurrence further suggests that both are associated with *HE* substrate growth and development.

## 4. Conclusions

Herein, we utilized SSF of *HE* to modify corn husk DF, revealing that the structural and physicochemical characteristics of the fermented corn husk SDF were superior to those of the pre-fermented corn husk SDF. These results indicate that SSF of *HE* can efficiently enhance corn husk SDF functional properties, which could benefit human health and have better applications. For instance, the modified corn husk SDF is incorporated into food products to enhance their intrinsic characteristics. Despite the potential drawbacks of incorporating dietary fiber into food products, technological advances and effective marketing strategies can facilitate the acceptance of modified dietary fiber foods and encourage their widespread use. The study also examined the fermentation kinetics of *HE* and found that the three types of extracellular enzyme activity increased and then decreased as fermentation time extended. Additionally, the content of total starch, reducing sugar, and IDF decreased overall, while the content of SDF showed an overall increase and then decreased. The changes in extracellular enzyme activity and nutrient composition during *HE* fermentation and the growth stage of mycelium were found to be closely linked. Additionally, a mutual promotion or inhibition relationship existed between extracellular enzyme activity and nutrient composition. In summary, fungal SSF is essential for modifying DF. The mechanism and metabolic pathway of fermentation-modified corn husk DF by *HE* can be investigated in the future. The relationship and mechanism between changes in extracellular enzyme vigor and nutrient composition during fermentation need further exploration.

## Data Availability

The original contributions presented in the study are included in the article, further inquiries can be directed to the corresponding author.

## Abbreviations

DF, Dietary fiber; *HE*, *Hericium erinaceus*; IDF, Insoluble dietary fiber; SDF, Soluble dietary fiber; SSF, Solid-state fermentation; PDP, Potassium dihydrogen phosphate; MSH, Magnesium sulfate heptahydrate; SEM, Scanning electron microscopy; FT-IR, Fourier transform infrared spectroscopy; WHC, Water holding capacity; RT, Room temperature; WSC, Expansion capacity; OHC, Oil holding capacity; OD, Optical density; CES, Crude enzyme solution; DNS, 3,5-dinitrosalicylic acid; CMC, Carboxymethylcellulase; HC, Hemicellulose
